# Omeprazole Induced Rapid Drug Reaction with Eosinophilia, Systemic Symptoms, and Cross-Reactivity in Delayed-Type Hypersensitivity Associated with Proton-Pump Inhibitors: A Case Report and Literature Review

**DOI:** 10.1155/2024/1317971

**Published:** 2024-01-03

**Authors:** Kanokkarn Pinyopornpanish, Kanokporn Pinyopornpanish, Kanokwan Pinyopornpanish, Juthipong Benjanuwattra, Putthapon Teepapan, Apinya Chungcharoenpanich, Wannada Laisuan

**Affiliations:** ^1^Division of Allergy, Immunology, and Rheumatology, Department of Medicine, Ramathibodi Hospital, Mahidol University, Bangkok, Thailand; ^2^Department of Medicine, Chiangmai University Hospital, Chiangmai, Thailand; ^3^Department of Family Medicine, Chiangmai University Hospital, Chiangmai, Thailand; ^4^Department of Internal Medicine, Texas Tech University Health Sciences Center, Lubbock, Texas, USA

## Abstract

**Background:**

Omeprazole, a proton pump inhibitor (PPI), is a widely used and generally safe agent for treating acid-related gastrointestinal conditions. However, drug reaction with eosinophilia and systemic symptoms (DRESSs) syndrome has been reported.

**Objectives:**

To report a case of omeprazole-induced rapid DRESS syndrome and to review the literature.

**Methods:**

Descriptive analysis of one new case and a case series from literature review.

**Results:**

We report a case of 82-year-old woman presenting with rapid-onset of DRESS syndrome. The condition was initially suspected to be caused by antibiotic, but the definite diagnosis was eventually omeprazole-induced DRESS syndrome as suggested by the enzyme-linked immune absorbent spot (ELISpot) assay along with the clinical picture. Previous literatures regarding cases of PPI-induced DRESS syndrome were pooled for descriptive analysis. Among 21 PPI cases pooled, esomeprazole was the most commonly implicated PPI (52.4%), followed by pantoprazole (19.1%), and omeprazole along with lansoprazole (both 14.3%). The issue of cross-reactivities amongst PPIs remains uncertain. Nonetheless, in situations in which a PPIs are deemed necessary, a prudent approach could be considering a switch to an alternative agent with distinct chemical structure.

**Conclusion:**

PPI is commonly used safely as an agent for acid-related gastrointestinal conditions. However, PPI-induced rapid DRESS syndrome can occur, particularly with prior exposure history. ELISpot is an in vitro test, useful in identifying the culprit agent in patients with delayed-type hypersensitivity reaction.

## 1. Background

Drug reaction with eosinophilia and systemic symptom (DRESS) is a severe and idiosyncratic drug reaction characterized by T cell-mediated hypersensitivity. The prevalence of DRESS has been estimated at 9.63 cases per 100,000 patients in Thailand and 2.18 per 100,000 in the United States [1, 2]. The development of DRESS involves a complex interaction between drug exposure, genetic predisposition, and viral reactivation, acting as risk factors and predisposing conditions [3]. Symptoms of DRESS typically emerge between 2 and 8 weeks after exposure to the causative agent [4]. However, individuals with prior exposure may have a shorter time duration between drug initiation and the onset of the reaction. Clinical presentation often includes an erythematous rash often accompanied by itching, facial edema, lymphadenopathy, hematologic abnormalities, visceral involvements, and high-grade fever [5, 6]. Even after discontinuation of the culprit agent, signs and symptoms of DRESS may persist with an average recovery period of 6–9 weeks [7]. Long-term sequalae following DRESS have been reported including autoimmune thyroiditis, fulminant type I diabetes, autoimmune hemolytic anemia, thrombotic thrombocytopenia purpura, and rheumatoid arthritis [3]. The mortality rate associated with DRESS is frequently reported to be approximately 3.8%–10%, which cardiac involvement in DRESS is associated with the highest mortality rate (45.2%–50%), followed by renal involvement (13%), and liver involvement (5%–10%) [8–12].

DRESS is commonly associated with various drugs, including anticonvulsants, allopurinol, sulfonamides, and antibiotics [2, 13]. According to RegiSCAR, aromatic anticonvulsants account for 35% of all cases of DRESS with carbamazepine the most frequent culprit agent. Allopurinol and sulfonamide are attributed to approximately 11%–18% along with 12% of cases, respectively. Although proton pump inhibitors (PPIs) rarely cause DRESS, they have been reported as the causative drug in 0.2%–0.3% of cases [14].

We report one case of omeprazole-induced DRESS syndrome and review the literature of PPI-induced DRESS syndrome cases to elucidate the clinical courses, culprit PPIs, diagnostic evaluations, and clinical outcomes associated with PPI-induced DRESS.

## 2. Case Presentation

**Figure 1:**
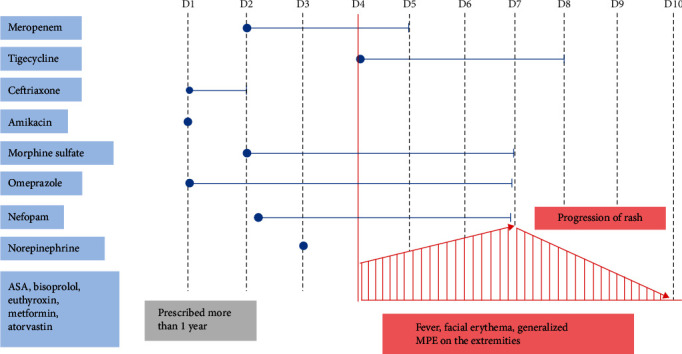
Timeline of drug administration and rash progression.

**Figure 2:**
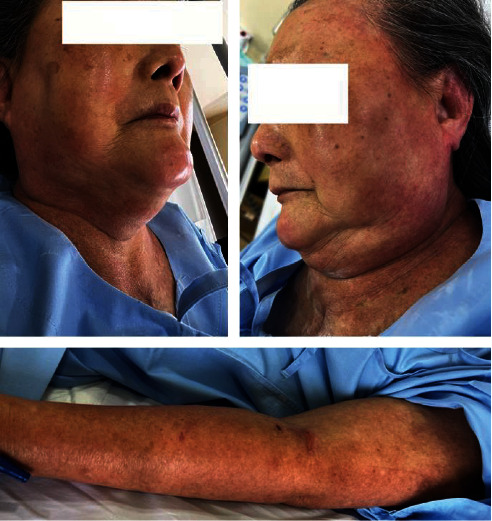
Skin manifestations suspected to be drug reaction with eosinophilia and systemic symptoms syndrome.

An 82-year-old Thai woman with a history of hypertension, type 2 diabetes mellitus, hypothyroidism, aortic valve replacement, and endometrial cancer presented with fever and abdominal pain for 3 days at the emergency department. The patient was admitted to the hospital and diagnosed with acute cholecystitis, for which she underwent open cholecystectomy the same day, and received postoperative ceftriaxone (2 g intravenously daily) and omeprazole (40 mg intravenously daily). On Day 2 of hospitalization, ceftriaxone was switched to meropenem due to suspected breakthrough infection. On Day 4 of hospitalization, she had a fever, facial erythema, and edema, as well as a generalized maculopapular rash on the extremities. The timeline of drugs administration and the appearance of skin manifestations is illustrated in Figures 1 and 2, respectively. Blood work revealed mild transaminitis (aspartate transaminase level, 61 U/L) and a slightly increased white blood cell count with eosinophilia (9,870 cells/mm^3^, eosinophils 8%). Renal function was normal (serum creatinine, 1.1 mg/dL). Based on the RegiSCAR scoring system [15], the patient received a total of two points, indicating a possible diagnosis of DRESS syndrome, including fever (≥38.5°C) = 0, eosinophilia = 1, extension of skin rash (>50%) = 1, and internal organ involved = 1.

At the time of the initial diagnosis of DRESS, meropenem was suspected to be the culprit agent due to prior reports indicating antibiotics may cause rapid DRESS [16]. Meropenem was consequently promptly discontinued. An allergist was consulted, and they suggested and expressed doubt that meropenem was the culprit agent, citing the short interval between exposure and the onset of symptoms. The progression of the skin rash was additionally documented, which could have been attributable to the persistence of the actual causative agent. Omeprazole was considered as the other likely culprit agent and was discontinued on Day 7 after admission. To confirm the suspected omeprazole-induced DRESS syndrome, an enzyme-linked immunosorbent spot (ELISpot) assay was performed, and the results supported this hypothesis (Table 1). As part of the treatment, the patient was prescribed oral prednisolone at a dose of 15 mg for 5 days. A histamine type-2 receptor antagonist (H2RA) was used for peptic ulcer prophylaxis in place of omeprazole. A gradual resolution of the skin rash, the eosinophilia, and the transaminitis were documented. Prednisolone was tapered at 5 mg every 3 days and then it was continued. On Day 10 of hospitalization prior to discharge, her clinical manifestations of DRESS syndrome had resolved including normalization of the eosinophil count.

### 2.1. Proton Pump Inhibitors Associated with DRESS: Literature Review

Case reports and related articles reporting on PPI-induced DRESS published between January 2005 and July 2023 were retrieved through keywords searching of the electronic database MEDLINE®/PubMed® (last search attempted 6 July 2023), as shown in Table 2.

A total of 21 cases were identified in which PPIs were associated with DRESS syndrome. Amongst these cases, the majority was female with mean ± SD age of 62.0 ± 11.3 years. The most commonly identified culprit drug was esomeprazole (11/21, 52.4%), followed by pantoprazole (4/21, 19.1%) and omeprazole along with lansoprazole (both 3/21, 14.3%).  Table 3 provides information on the clinical and internal organ involvement observed in these cases. Cross-reactivities between each case's reported culprit PPI and other PPIs are shown in Table 4.

## 3. Discussion

In the present case report, we have identified a possible case of DRESS with symptoms manifesting merely 4 days after drug exposure. The patient's syndromic presentation was a skin rash, eosinophilia, hepatitis, and acute kidney injury although lymphadenopathy was not observed. Initially, our suspicions fell on meropenem as the causative drugs, prompting us to discontinue its use. However, the progression of the rash and eosinophilia persisted, prompting us to redirect our attention to omeprazole as the potential culprit responsible for the DRESS syndrome in the present case. Subsequently, upon discontinuation of omeprazole, we observed the resolution of skin rash and internal organ involvement.

In retrospective study conducted by Soria et al. [16], a cohort of DRESS syndrome cases with short latency was investigated. Antibiotics and iodinated contrast media emerged as potential causative drugs with a mean ± SD onset of symptoms at approximately 7.3 ± 4.0 days after drug exposure. Notably, the short latency group exhibited a significantly lower incidence of lymphadenopathy compared to cases with symptoms appearing greater or equal to 15 days after exposure to the culprit agent. As per our review, the latency period of DRESS from the PPI ranges from 8 to 53 days. However, in some studies, the latency of DRESS could be 2–180 days [27].

Although omeprazole was not identified as a common culprit PPI causing the rapid onset of DRESS syndrome in the aforementioned study [16], it is worth considering the potential role of prior exposure to PPIs, such as omeprazole. PPIs are extensively utilized in both outpatient and in-patient settings for the treatment of gastric ulcers. The consideration gains support from our understanding of DRESS pathogenesis, which involves T cell-mediated hypersensitivity [28]. The presence of preexisting memory T cells could potentially contribute to the accelerated onset of DRESS syndrome observed in the present reported case following exposure to omeprazole.

Currently, clinical diagnosis remains the gold standard for diagnosis of DRESS syndrome. Various factors including the time from drug initiation to the development of delayed reaction, clinical presentation, and the drugs class in relation to specific drug reactions should be evaluated to identify the culprit drug while testing for delayed reactions has limited evidence and is often based on small case series without drug challenge. Patch tests are used for investigating the culprit agents and underlying hypersensitivity mechanisms, but they have low sensitivity (32%) and require good skin conditions [29]. Instead of patch tests, ELISpot assay and lymphocyte stimulation test can be employed albeit in research laboratories more than routine clinical practice. However, it is important to note that in vitro testing alone cannot definitively confirm or rule out a drug reaction, and clinical history remains the reference standard [30].

**Figure 3:**
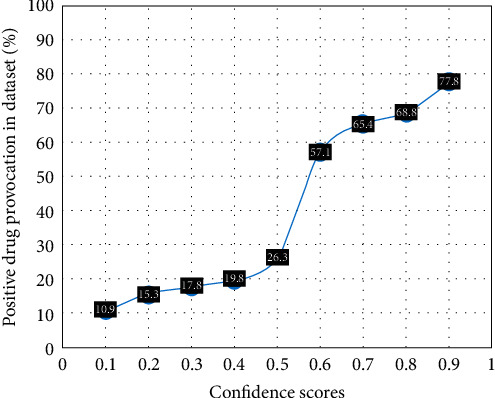
Predictive model conceived from 1,024 ELISpot assays and 165 drug challenges. *Source*: Hipaa's [32] study.

As discussed with the patient, the ELISpot assay exhibits the highest sensitivity and specificity in cases of DRESS, with a sensitivity of 61% and specificity of 97% [16]. However, the sensitivity and specificity of the ELISpot depend on factors such as Naranjo score, drug allergy phenotype, type of suspected drug, and underlying disease, as mentioned in Chongpison et al.'s [31] study. The confidence score calculated by machine learning for omeprazole was 0.80 (percentage of positive challenge 68.8), while for meropenem and ceftriaxone, it was 0.15 (percentage of positive challenge around 13.1) (as Figure 3). This suggests that she might be experiencing DRESS due to omeprazole rather than an antibiotic. In this case, our plan of action is to conduct skin tests and provocation tests using ceftriaxone and meropenem in an outpatient setting. However, on the next visit, she was receiving ceftriaxone without same reaction.

Previous studies have reported promising results for the ELISpot assay in identifying the culprit agent with a sensitivity of 70% in DRESS syndrome patients [33–35]. Supplementation with anti-PD-1 significantly increased the frequencies of drug-induced IFN-*γ*- releasing cells. A positive ELISpot assay result was determined by frequencies of drug-induced IFN-*γ* reaching 17.2 SFU/10^6^ PBMCs [36]. In our case, the ELISpot assay using f omeprazole and anti-PD-1at 5 ng/mL produced positive results with frequencies of drug-induced IFN-*γ* of 24 SFU/10^6^ PBMCs. A previous study nonetheless reported a specificity of 82.9% for the ELISpot assay in the detection of beta-lactam hypersensitivity [37].

To prevent repeated drug reactions, particularly in severe cutaneous adverse reactions, it is imperative to carefully evaluate the potential for cross-reactivity between the implicated drugs and other agents. However, the issue of cross-reactivity among PPIs remains a matter of ongoing debate due to the lack of a definite understanding of the underlying mechanism. In a study conducted in Taiwan involving 69 cases of PPI-related delayed-type hypersensitivity, 10 cases of DRESS syndrome were identified. Among these cases, esomeprazole emerged as the PPI most frequently associated with delayed-type hypersensitivity reaction. Interestingly, alternative PPIs with distinct chemical structures were administered to 15 patients, resulting in improved tolerance. These findings suggest that individuals with a history of PPI-induced delayed-type hypersensitivity may benefit from avoiding PPIs that share similar chemical structures [22]. It is noteworthy, however, that even drugs with disparate chemical structures, such as lansoprazole and omeprazole, can still demonstrate cross-reactivity in certain instances. Clinicians should, therefore, be cautious when managing such patients [22].

The present study has a limitation. It lacked viral studies including assessments of HHV6, HHV7, CMV, and EBV viral loads. The present study nonetheless encompassed clinical evaluations and in vitro investigations, such as ELISpot assay.

In conclusion, clinicians must not overlook the possibility of PPI-induced DRESS syndrome. Identifying the culprit agent is essential for effective long-term management, particularly in individuals requiring acid suppression. Non-PPI treatment options can be considered due to limited data on cross-reactivity. In cases in which a PPI is deemed necessary, switching to an alternative with distinct chemical structure, coupled with long-term follow-up, and appropriate drug labeling to prevent future exposure may, however, be advisable.

## Figures and Tables

**Table 1:** ELISpot results.

Allergen/drugs	Dose	SFU/10^6^ PBMC
		IFN-*γ* ELISpot
Ceftriaxone	40 *μ*g/mL	0
Ceftriaxone	200 *μ*g/mL	0
Ceftriaxone + anti-PD-L1	40 *μ*g/mL	0
Ceftriaxone + anti-PD-L1	200 *μ*g/mL	0
Meropenem	40 *μ*g/mL	0
Meropenem	200 *μ*g/mL	0
Meropenem + anti-PD-L1	40 *μ*g/mL	0
Meropenem + anti-PD-L1	200 *μ*g/mL	0
Omeprazole	1 *μ*g/mL	0
Omeprazole^a^	5 ng/mL	8
Omeprazole + anti-PD-L1	1 *μ*g/mL	16
Omeprazole + anti-PD-L1	5 ng/mL	24
PHA	5 mg/mL	3,388

*Note*: ^a^Positive result for ELISpot activation at an omeprazole concentration of 5 ng/mL.

**Table 2:** Publication reporting proton pump inhibitors associated with DRESS.

Reference	PPI drug	Age (year)	Sex	Onset (day)	Presentation	Abnormal tests	Remark
Carboni et al. [17]	Esomeprazole	41	F	20	(i) Erythematous and itching skin reaction with maculopapular diffuse lesions, followed by desquamation (ii) Fever	(i) Eosinophilia (ii) Hepatitis	Patch tests were positive with esomeprazole, omeprazole, and pantoprazole

Barbaud et al. [18]	Esomeprazole	64	F	NA	NA	NA	Patch test positive
	Esomeprazole	60	M	NA	NA	NA	Patch test positive
	Pantoprazole	31	F	NA	NA	NA	Patch test positive
	Pantoprazole	51	F	NA	NA	NA	Patch test negative
	Lansoprazole	75	F	NA	NA	NA	Patch test negative

Bourneau-Martin et al. [19]	Omeprazole	88	F	53	(i) Maculopapular exanthema on the trunk and the face converging with wide patches with purpuric lesions (ii) Fever (iii) Lymphadenopathy	(i) Creatinine rising (ii) Eosinophilia (iii) Lymphocytosis	Skin biopsy: inflammatory cell infiltrate rich in neutrophils and lymphocytes

Zaiem et al. [20]	Esomeprazole	84	F	8	(i) Fever (ii) Maculopapular rash	(i) Creatinine rising (ii) Eosinophilia (iii) Hepatitis	Dead

Uppalapati and Koneru [21]	Lansoprazole	40	F	30	(i) Pruritis (ii) Generalized maculopapular rash (iii) Palmar erythema	(i) Creatinine rising Eosinophilia (ii) Hyperbilirubinemia	Skin biopsy showed perivascular inflammatory infiltrate with elevated eosinophils

Lin et al. [22]	Pantoprazole (*n* = 2) Esomeprazole (*n* = 7) Lansoprazole (*n* = 1)	61.9 ± 8.0	NA	27.2 ± 19.1	(i) Skin rash > 50% of the BSA (ii) At least one internal organ abnormality (iii) One blood work abnormality (iv) Lymphadenopathy (v) Fever > 38.5°C	(i) Atypical lymphocyte (*n* = 6) (ii) Creatinine rising (*n* = 3) (iii) Eosinophilia (*n* = 8) (iv) Hepatitis (*n* = 8) (v) Pneumonitis (*n* = 3)	—

He et al. [23]	Omeprazole	66	F	14	(i) Pruritus (ii) Desquamation (iii) Erythema multiforme (iv) Fever	(i) Creatinine rising (ii) Eosinophilia	Renal biopsy: chronic tubular interstitial inflammation diffuse atrophy of the renal tubular epithelium, extensive lymphocytic infiltration of the renal interstitial, and occasionally eosinophils

Present study's case report	Omeprazole	82	F	4	(i) Facial erythema (ii) Facial edema (iii) Generalized maculopapular rash (iv) Fever	(i) Hepatitis (ii) Eosinophilia	ELISpot to omeprazole positive

*Note*: PPI, proton pump inhibitor; NA, non-applicable; F, female; M, male; BSA, body surface area; ELISpot, enzyme-linked immunosorbent spot.

**Table 3:** Clinical presentation, internal organs involvement, and laboratory abnormalities in proton pump inhibitors induced DRESS in 16 patients.

Characteristics in PPIs-induced DRESS	Percentage affected (*n* = 16)
Skin rash involve > 50% BSA	100
Fever	93.8
Eosinophilia	87.5
Hepatitis	68.8
Creatinine rising	43.8
Pneumonitis	18.8

*Note*: BSA, body surface area; DRESS, drug reaction with eosinophilia and systemic symptoms syndrome.

**Table 4:** Cross-reactivity among proton-pump inhibitors in delayed type hypersensitivity.

Causative PPI/phenotype	Omeprazole	Pantoprazole	Esomeprazole	Lansoprazole	Dexlansoprazole	Rabeprazole
Omeprazole	—	—	—	—	—	—

Pantoprazole/airborne contact dermatitis Pantoprazole/SJS	+P [24]	—	+DPT [22]	—	—	—

Esomeprazole/delayed urticaria esomeprazole/DRESS esomeprazole/MPE esomeprazole/SJS/TEN	−SPT [25] +P [17]	+SPT [25] +P [17]	—	−DPT [25] −DPT [22] −DPT [22] −DPT [22]	−DPT [22]	−DPT [22]

Lansoprazole/pruritic papules lansoprazole/MPE lansoprazole/SJS/TEN lansoprazole/DRESS	+P [26]	−DPT [22]	−DPT [22] −DPT [22]	—	—	+DPT [22]

Dexlansoprazole/MPE	—	—	−DPT [22]	—	—	+DPT [22]
Rabeprazole	—	—	—	—	—	—

*Note*: DPT, drug provocation test; P, patch test; PPI, proton pump inhibitor; SPT, skin prick test; −, negative result; +, positive result; MPE, maculopapular eruption; SJS/TEN, Stevens–Johnson syndrome/toxic epidermal necrolysis; DRESS, drug reaction with eosinophilia and systemic symptom.

## Data Availability

The data used to support the finding of this study are included within the article.
